# Optimization and standardization of mNGS-based procedures for the diagnosis of *Mycoplasma* periprosthetic joint infection: A novel diagnostic strategy for rare bacterial periprosthetic joint infection

**DOI:** 10.3389/fcimb.2023.1089919

**Published:** 2023-03-01

**Authors:** Yuanqing Cai, Haiqi Ding, Xiaoqing Chen, Yang Chen, Changyu Huang, Chaofan Zhang, Zida Huang, Ying Huang, Wenbo Li, Wenming Zhang, Xinyu Fang

**Affiliations:** ^1^ Department of Orthopedics, National Regional Medical Center, Binhai Campus of the First Affiliated Hospital, Fujian Medical University, Fuzhou, Fujian, China; ^2^ Department of Orthopedics, The Second Affiliated Hospital of Xi’an Jiaotong University, Xi’an, Shaanxi, China; ^3^ Department of Orthopedics, The First Affiliated Hospital, Fujian Medical University, Fuzhou, Fujian, China; ^4^ Department of Orthopedics, Quanzhou First Hospital Affiliated to Fujian Medical University, Quanzhou, China

**Keywords:** *Mycoplasma*, periprosthetic joint infection, metagenomic next-generation sequencing, procedure, rare bacteria

## Abstract

**Introduction:**

The diagnosis of *Mycoplasma* periprosthetic joint infection (PJI) is rather difficult due to its rarity and difficult in isolation, there are not standardized diagnostic procedure for *Mycoplasma* PJI presently. This study aimed to reported a metagenomic next-generation sequencing (mNGS)-based diagnostic strategy for *Mycoplasma* PJI.

**Methods:**

In the present study, we have reported the largest number of *Mycoplasma* PJI that were precisely diagnosed by mNGS and verified by optimized microbial culture methods and (or) 16S PCR polymerase chain reaction (PCR).

**Results:**

The positive rate of optimized microbial culture methods and 16S PCR in the detection of *Mycoplasma* PJI was 57.14% and 71.43%, respectively. The infections were well controlled by targeted treatment in all cases.

**Conclusion:**

The standardized and optimized procedure based on mNGS presented in this study is useful for the diagnosis of *Mycoplasma* PJI, which might also be provided as a novel diagnostic strategy for rare bacterial PJI.

## Introduction

Periprosthetic joint infection (PJI) is one of the serious complications after joint arthroplasty, multiple debridement operations and extended period antibiotic treatment are need (Rajput et al., 2022). PJI is often caused by gram positive or negative bacteria such as *Staphylococcus aureus*, *Staphylococcus epidermidis*, *Escherichia coli* and *Pseudomonas aeruginosa* through surgical incision infection or hematogenous infection (Bjerke-Kroll et al., 2014; McCulloch et al., 2022). With the increasing number of patients undergoing joint arthroplasty, as well as the use of antibiotics and the increase of medical-related invasive procedures (such as arthrocentesis, urinary catheterization, etc.), PJI caused by rare bacteria are becoming more common (Escolà-Vergé et al., 2018; Spanyer et al., 2018; Brzezinski et al., 2021). Rare bacterial PJI might be easily missed diagnosed because they could not be effectively isolated, therefore the antibiotics used might not cover pathogenic bacteria, resulting in persistent infection. Therefore, it is necessary to establish a standardized and optimized diagnostic procedure for PJI caused by such rare bacteria.

*Mycoplasma* is the smallest prokaryotic organisms, widely exists in people and animals, and most of them are not pathogenic. *Mycoplasma* is a common causative agent in pulmonary and genitourinary tract infections, so clinicians usually use microbial culture and polymerase chain reaction (PCR) for pathogen detection (Volsky, 1990; Nadal et al., 2001). However, the incidence rate of *Mycoplasma* PJI is relatively low, it could be easily overlooked by surgeons. In recent years, *Mycoplasma* has been increasingly reported in osteoarticular infection (Chen et al., 2020) and PJI, especially those caused by *Mycoplasma hominis*, but mostly as case reports and incidental positive cultures (Qiu et al., 2017; Rieber et al., 2019; Xiang and Lu, 2019; He et al., 2020; Muramatsu et al., 2022). Therefore, it is necessary to determine whether *Mycoplasma* PJI cases are underdiagnosed, or whether these cases are false positives caused by specimen contamination. A more efficient, optimal, and standardized diagnostic procedure is needed.

Our center has been applying metagenomic next-generation sequencing (mNGS), a new molecular diagnostic tool, in the diagnosis of osteoarticular infection for long time (Huang et al., 2019; Cai et al., 2020; Fang et al., 2021; Fang et al., 2022). It is a culture-independent, unbiased pathogen detection tool which offers additional advantages in the detection of pathogenic bacteria that cannot be isolated by routine microbial culture methods. By this method, we have detected *Mycoplasma* from many PJI cases (Fang et al., 2021). In order to eliminate the clinician’s doubts about whether *Mycoplasma* detected by mNGS is a real pathogenic bacterium, based on the largest number of *Mycoplasma* PJI cases reported in this study, we have established a standardized and novel diagnosis procedure of *Mycoplasma* PJI, and this procedure can be generalized to the diagnosis of other rare bacterial PJI.

## Materials and methods

### Participants

This study included the suspected PJI patients who were admitted in our hospital from 2015 to 2021. The diagnosis of PJI was based on Musculoskeletal Infection Association (MSIS) criteria: at least one of following three criteria should be met: 1) sinus tract was communicating with the prostate; 2) pathogen was isolated by culture from two separate tissue or fluid samples obtained from the affected prosthetic joint; and 3) patient met four of the following six criteria: (a) elevated ESR and CRP; (b) elevated synovial fluid WBC count; (c) elevated synovial fluid neutrophil percentage; (d) presence of purulence in the affected joint; (e) isolation of a microorganism in one periprosthetic tissue or fluid culture; and (f) > 5 neutrophils per high-powered field in 5 high-power fields observed from histologic analysis of periprosthetic tissue at ×400 magnification. Inclusion criteria were as follows: 1) patients who were suspected of *Mycoplasma* PJI according to microbial culture and mNGS results; 2) patients with complete medical data. Exclusion criteria were as follows: 1) insufficient clinical and laboratory data to diagnose PJI; 2) contamination or suspected contamination of samples; 3) infections at other sites or in the body.

### Routine microbial culture

Specimens were collected and transported to the microbiology laboratory within 30 minutes. For the synovial fluid, 100μL was inoculated on the blood agar, 1000μL was used for mNGS, and the rest was injected into the blood culture bottles for microbial culture, when the culture results were positive, they were transferred to the blood agar. For the tissue specimens, they were ground in sterile tryptic soy broth Ep tubes and then the homogenate was inoculated on the blood agar. For the removed prosthesis, they were immersed with sterile saline, sonicated, supernatant was removed after centrifugation, resuspended with sterile saline, and then injected into blood culture bottles for microbial culture.

### mNGS

The mNGS was performed according to previous describe methods (Cai et al., 2020; Huang et al., 2020; Fang et al., 2021). In brief, the procedure including: 1) nucleic acid extraction: the total DNA was extracted by TIANamp Micro DNA Kit (DP316, Tiangen Company, China) from homogenate or synovial fluid; 2) library construction: after DNA was randomly fragmented into 200-300bp nucleic acid fragments, constructed DNA libraries by end repair, specific splice ligation, purification, PCR amplification and cyclization reactions to generate single-stranded DNA loops. 3) sequencing: the DNA libraries were processed for 50 bp single-end sequenced by BGISEQ-500 platform (Huada Zhizhi, China). 4) Bioinformatics analysis: remove the data with low quality and less than 35bp from the raw sequencing data, and then remove the Human reference genome sequence (Hg19) by Burrows-Wheeler alignment (http://biobwa.sourceforge.net). The remaining sequence alignment to the microbial reference database, obtained from National Center for Biotechnology Information (NCBI) and classified as viruses, fungi, bacteria, parasites, etc. 5) Interpretation of mNGS results: the interpretation was conducted by the PJI panel, including at least one senior microbiologist, infectious disease expert and orthopedic expert dedicated to PJI research.

### Verification by optimized culture methods

For PJI cases with *Mycoplasma* reported by mNGS preoperatively, liquid combined with solid culture method (optimized culture method) was used for validation after sufficient samples were collected intraoperatively (Fang et al., 2021). Liquid sample were cultured by *Mycoplasma* ICS Kitliquid (Zhuhai Livzon Reagent Co., Ltd.). In the liquid medium, micropores were closed with paraffin, after which samples were immersed and cultured at 37°C with 5% CO_2_, and the results were recorded at 24, 48 and 72 hour (h). When the bacteria concentration ≥ 10^4^ cfu/ml, the micropores became red and were confirmed to be positive; for solid medium, samples were directly incubated in elective solid medium and cultured at 37°C in a 5% CO_2_ incubator for 24, 48, and 72 h, observed under low magnification (10×10) to confirm that the colonies were *Mycoplasma* if they were small and fried egg-like.

### Verification by 16S PCR

For PJI cases with *Mycoplasma* reported by mNGS preoperative, sufficient samples were collected intraoperatively and verified by 16S PCR. Briefly, 200 μL specimen was placed in a disposable 2 mL sterile centrifuge tube, and the total DNA was extracted by DNeasyBlood & Tissue kit (QIAGEN, Germany) as per manufacturer’s instructions. Digested with protease K for 4 h, and the extracted DNA was dissolved in 100 μL double distilled deionized water. The bacterial/fungal genomic DNA Mix was used as the positive control. The PCR conditions are: pre-denaturation at 94°C for 2 min, denaturation at 94°C for 20 s, annealing at 55°C for 30 s and extension at 72°C, with 30 cycles, extension at 72°C for 10 min and preservation at 4°C. The product was subjected to agarose gel electrophoresis and judged as negative amplification if no band was shown or if the band was not recognized by two PCR lab technicians. If positive, the PCR products were sent for sequencing and analysis (Shanghai Tianhao Biotechnology Co., Ltd.), and the sequencing results alignment to Gen Bank to determine the species.

### Verification by targeted treatment

For acute PJI, surgical treatment was performed with debridement, antibiotic irrigation and implant retention (DAIR), while for chronic PJI, two-stage revision was selected. After surgical treatment, PJI cases received intravenous antibiotic treatment for 2 weeks and oral antibiotic therapy for 1-3 months after discharge. PJI panel re-formulated the antibiotics regimen for *Mycoplasma* based on the results of mNGS and other verification methods, combined with the clinical efficacies, and the accuracy of mNGS and other verification methods were also confirmed according to the clinical efficacies of *Mycoplasma* targeted treatment. All these cases were followed up regularly and the clinical data like CRP, ESR, infection recurrence and the incidence of antibiotic-related complications were recorded.

## Results

### Demographic characteristics

A total of 7 *Mycoplasma* PJI cases were included in this study, which was the largest sample of *Mycoplasma* PJI cases so far (**Table 1** for details). The average age was (72.57 ± 14.06) years old, including 4 male cases and 3 female cases, there were 4 knee PJI cases and 3 hip PJI cases. Interestingly, one of them was a case with PJI in bilateral knee. Among these patients, 5 cases were PJI infected by single bacterium, and among the other two patients, case 1 was mixed infection, case 2 was mixed infection in the right knee and single bacterium infection in left knee. In terms of subtypes of *Mycoplasma*, most patients were infected with *Mycoplasma hominis*, while case 1 with *Mycoplasma hyorhinis*, and case 7 with *Ureaplasma urealyticum*. Most of these patients received the two-stage revision surgery, and only the case with bilateral knee PJI were treated with DAIR.

**Table 1:** Periprosthetic joint infection cases caused by *Mycoplasma*.

No.	Age	Sex	Site	Routine microbial culture	mNGS	Treatment	Antibiotics
1	77	F	Hip	*Staphylococcus epidermidis*	*Staphylococcus epidermidis/* *Mycoplasma hyorhinis*	Two-stage revision	Levofloxacin+ Doxycycline
2	88	M	Knee (R)	*Staphylococcus aureus*	*Staphylococcus aureus/ Mycoplasma hominis*	DAIR	Levofloxacin+ Doxycycline
	88	M	Knee (L)	Negative	*Mycoplasma hominis*	DAIR	Doxycycline
3	87	M	Hip	Negative	*Mycoplasma hominis*	Two-stage revision	Doxycycline
4	52	M	Hip	Negative	*Mycoplasma hominis*	Two-stage revision	Ciprofloxacin +Tetracycline
5	65	F	Knee	Negative	*Mycoplasma hominis*	Two-stage revision	Doxycycline
6	59	F	Knee	Negative	*Mycoplasma hominis*	Two-stage revision	Tetracycline
7	80	F	Knee	Negative	*Ureaplasma urealyticum*	Two-stage revision	Doxycycline

mNGS, metagenomic next-generation sequencing; R, right; L, left; DAIR, debridement antibiotics irrigation and implant retention.

### The positive rates of various *Mycoplasma* detection methods

In this study, all cases were precisely diagnosed by mNGS. Although some case reports demonstrated that *Mycoplasma* could be isolated by conventional microbiological culture methods, however, *Mycoplasma* could not be isolated by routine microbial culture methods in this study. Based on mNGS results preoperatively, both the optimized microbial culture methods and 16S PCR were used to verify the accuracy of mNGS. The results showed that although the optimized microbial culture method work in the *Mycoplasma* isolation, only 4 cases (case 2(right),3,4,6) could be successfully isolated with a positive rate of about 57.14% (4/7), while the positive rate further increased to 71.43% (5/7) by 16S PCR (Case 2 (left), 3, 4,6,7), but was still lower than that of mNGS. However, the relatively small number of *Mycoplasma* PJI cases and the lack of controls prevented comparison of positivity rate between mNGS and other validation methods.

### Clinical efficacies of targeted treatment

Based on mNGS, routine and optimized microbial culture and 16S PCR results, PJI panel judged whether *Mycoplasma* was the true pathogenic bacteria combined with the patient’s medical history, symptoms, and other examination results. In this study, although *Mycoplasma* was only detected from 5 patients (case 2, 3, 4, 6, 7), the panel still diagnosed the other 2 patients as *Mycoplasma* PJI (case 5) or *Mycoplasma* complicated with other bacterial infections PJI (case 1) according to the poor clinical efficacies of routine microbial culture results oriented or empirical antibiotics treatment. Therefore, after surgical treatment (DAIR or two-stage revision), all patients received doxycycline or tetracycline for anti-infection treatment. All patients responded well to the targeted treatment and eventually controlled the infection. At the last follow-up, all patients had good joint mobility and functional scores, no patients need surgical debridement or long-term antibacterial therapy due to recurrence of infection, only one patient (case 1) developed antibiotic-related complications (slight allergic reaction), which was relieved after symptomatic treatment. Thus, based on the results of mNGS and various validation methods, *Mycoplasma* PJI can be successfully diagnosed and cured with targeted treatment.

### Cases information

Case 1 was a 77-year-old woman who underwent total hip arthroplasty (THA) for femoral neck fracture 5 years ago. Her hip pain had worsened for about 2 weeks, and she was admitted with a C-reactive protein (CRP) of 7. 38 mg/L and an erythrocyte sedimentation rate (ESR) of 36 mm/h. A synovial fluid aspiration and test was performed preoperatively, the synovial fluid white blood cell (SF-WBC) was 56367×10^6^/L and the synovial fluid polymorphonuclear neutrophils (SF-PMN) percentage was 89.9%. To better identify the pathogenic organisms, routine microbial culture and mNGS were both performed preoperatively. About 48 hours later, the mNGS results indicated that it was a multiple infection caused by *Staphylococcus epidermidis* (*S. epidermidis*) and *Mycoplasma hyorhinis*, while microbial culture and 16S PCR only showed the potential infection by *S. epidermidis*. Unfortunately, CRP and ESR remained at high levels and the infection could not be controlled after microbial culture results oriented antibiotics treatment. Based on symptoms, physical examination and medical history, the PJI panel reconsidered that the *Mycoplasma hyorhinis* was also the true pathogen rather than background bacteria. Therefore, a two-stage revision was performed and a modified antibiotic regimen (levofloxacin + doxycycline) was adopted, and finally the inflammatory indexes decreased to normal and the infection was well controlled.

Case 2 was an 88-year-old male who received total knee arthroplasty (TKA) on the right knee due to osteoarthritis. Five days post-operation, his right knee had been swollen and painful, and a small amount of pus was exuded from the incision on admission. Laboratory tests showed elevated CRP (>90 mg/L), ESR (120 mm/h), SF-WBC (23177×10^6^/L) and SF-PMN% (90.8%), a diagnosis of acute PJI was made. In order to timely identify the pathogen, routine microbial culture and mNGS were both performed preoperatively. mNGS showed *Staphylococcus aureus* (*S. aureus*) and *Mycoplasma hominis* infection, while the culture only indicated *S. aureus*. The diagnosis of acute PJI was made and DAIR was performed. Samples were also collected intraoperatively for optimized culture and 16S PCR, the 16S PCR results was negative while optimized microbial culture indicated the infection by *Mycoplasma hominis*. Therefore, this case was treated as mixed infection with a combination antibiotic regimen (levofloxacin+ doxycycline) determined by the PJI panel for about six weeks and the infection was controlled well. One year later, the patient underwent a total knee arthroplasty (TKA) on another knee for severe osteoarthritis. Unfortunately, his left knee was swollen and painful again after surgery, with elevated inflammatory indexes on admission. At this time, mNGS showed that this might be a *Mycoplasma hominis* PJI case, while the microbial culture results were negative. Interestingly, 16S PCR also indicated that the pathogenic bacteria might be *Mycoplasma hominis*, validating the accuracy of mNGS. Therefore, the patient was treated with DAIR and doxycycline, and the clinical outcome was satisfactory.

**Figure 1:**
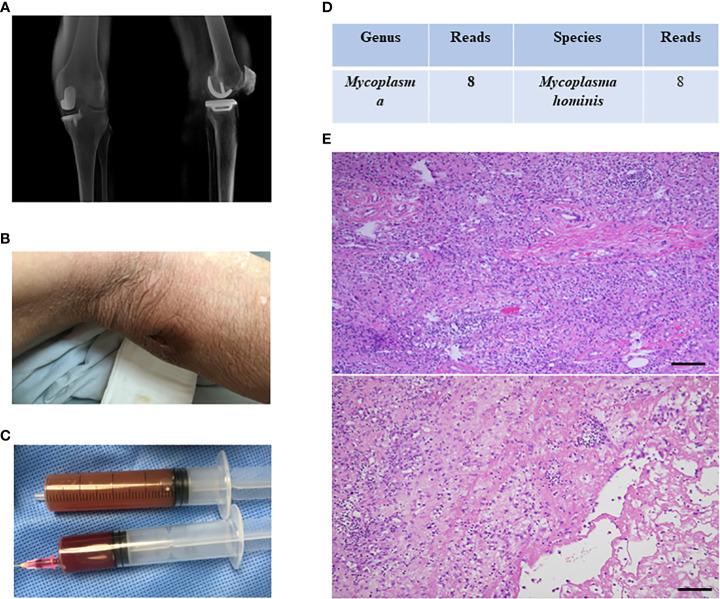
The clinical data of case 5. **(A)** the imaging data of case 4; **(B)** there was a sinus tract behind the knee; **(C)** purulent synovial fluid was obtained from preoperative aspiration; **(D)** mNGS indicated *Mycoplasma hominis* infection; **(E)** intraoperative pathological test showed there were a large amount of neutrophils in periprosthetic tissues.

Case 3-7 were all PJI cases caused by *Mycoplasma* (case 3-6 were *Mycoplasma hominis*; case 7 was *Ureaplasma urealyticum*). The pathogens were identified by mNGS and routine microbial culture results were all negative, but optimized microbial culture results were positive in cases 3,4, 6, and 16S PCR results were positive in case 3,4,6,7. Among these cases, case 5 was a patient who underwent UKA on the left knee 4 years ago (**Figure 1A**) and was admitted with knee pain for about 1 month and a sinus tract behind the knee (**Figure 1B**). Purulent synovial fluid was obtained from preoperative aspiration (**Figure 1C**). *Mycoplasma hominis* was reported as a pathogen for preoperative mNGS (**Figure 1D**), but routine microbial culture results were negative. The verification by optimized microbial culture and 16S PCR were both negative. Two-stage revision was performed for this case, and there were a large amount of neutrophils in periprosthetic tissues showed by intraoperative pathological test (**Figure 1E**). And this case was treated as culture negative PJI with empiric antibiotics regimen but resulting in infection control failure. Thus, the PJI panel concluded that the *Mycoplasma hominis* was the true pathogenic bacteria and doxycycline was used to control the infection with good clinical outcome.

### The optimized and standardized mNGS based diagnosis procedure for *Mycoplasma* PJI

**Figure 2:**
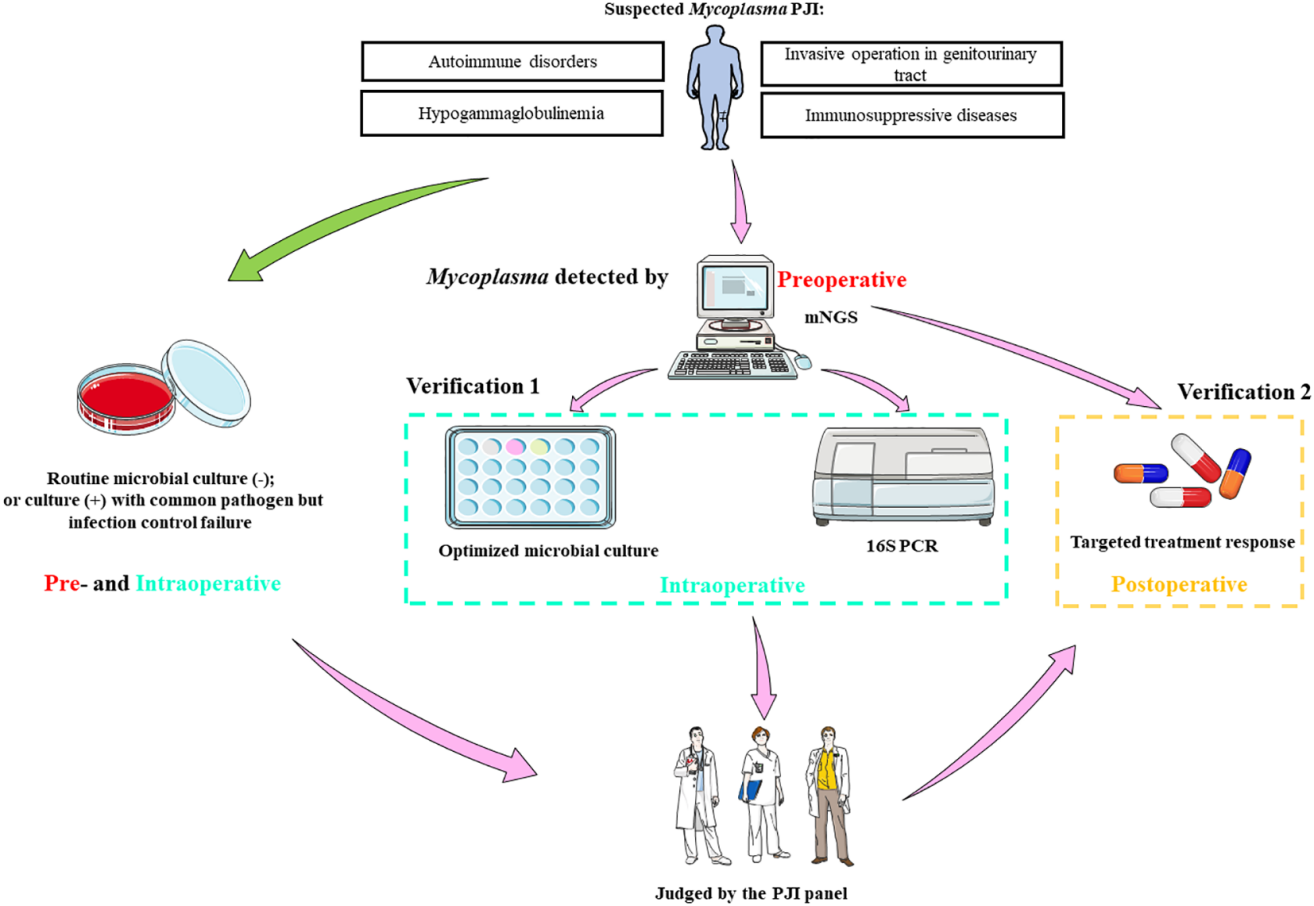
The optimized and standardized procedure based on mNGS for Mycoplasma PJI. mNGS, metagenomic next-generation sequencing; PCR, polymerase chain reaction; PJI, periprosthetic joint infection; -, microbial culture negative; +, microbial culture positive. Parts of the figure were drawn by using pictures from Servier Medical Art. Servier Medical Art by Servier is licensed under a Creative Commons Attribution 3.0 Unported License (https://creativecommons.org/licenses/by/3.0/).

Based on above results, we concluded an mNGS based optimized and standardized diagnosis procedure for *Mycoplasma* PJI (**Figure 2**). Patients with autoimmune diseases, hypogammaglobulinemia, other potential immunosuppression, and recent invasive operation in genitourinary tract are risk factors of *Mycoplasma* PJI. At our center, preoperative joint aspiration fluid of mNGS was usually used as an auxiliary diagnostic method, these results would be informative once the routine microbial culture results of specimen collected intraoperatively were negative, or failure in infection control with empirical antibiotic treatment even the culture results were positive. Thus, *Mycoplasma* detected by mNGS preoperatively could provide a good reference for clinicians. Because sufficient samples could be easily obtained intraoperatively, various methods can be used to validate the mNGS results. These methods include: 1) optimized microbial culture method; 2) after DNA extraction from samples, 16s PCR amplification and sequencing could be performed. Finally, the PJI panel comprehensively judged the results of these methods, and decided whether to modify the antibiotic regimens according to *Mycoplasma* reported by mNGS based on the therapeutic effect of the primary antibiotics. The final diagnosis of *Mycoplasma* PJI was made by the clinical efficacy of targeted treatment (**Figure 2**). It should be note that this procedure might also be generalized to PJI caused by rare bacteria.

## Discussion

*Mycoplasma* is one of the normal colonized flora of genitourinary tract and is usually non-pathogenic (Ahmed et al., 2021). However, previous literature reported that *Mycoplasma* PJI after arthroplasty was transmitted to the surgical site primarily through the bloodstream (Xiang and Lu, 2019). The most common reason for this was that urethral catheterization during arthroplasty disrupted the genital tract mucosa and *Mycoplasma* entered the bloodstream through the broken mucosa, causing bacteremia, which was then transmitted through the bloodstream to the joint undergoing arthroplasty (Xiang and Lu, 2019). Although *Mycoplasma* was less pathogenic, it can lead to serious complications such as sepsis and even death (MacKenzie et al., 2010; Xiang and Lu, 2019). A patient was previously reported to be die of disseminated infection of *Mycoplasma hominis* and *Ureaplasma parvum* in the hip after arthroplasty (MacKenzie et al., 2010). Therefore, improving the detection rate of pathogenic bacteria in *Mycoplasma* PJI was clinically important to improve treatment outcomes and avoid serious complications.

mNGS can detect all pathogenic bacteria in samples without bias, and has significant advantages in detecting some rare bacteria, fastidious bacteria and other rare pathogenic bacteria (Thoendel et al., 2017; He et al., 2020). Wang et al. (Wang et al., 2021) reported a case of knee PJI infected by *Mycoplasma hominis*, in which Wang et al. innovatively introduced mNGS to detect pathogenic bacteria (Wang et al., 2021). mNGS results indicated that the pathogenic bacteria might be *Mycoplasma hominis* for twice. Therefore, this case was treated with anti-*Mycoplasma hominis* antibiotics, and finally got good clinical efficacy. In this study, we successfully detected *Mycoplasma* in all seven patients with mNGS and treated them with antibiotics based on the results of mNGS, which eventually controlled the infection. Although the number of cases of *Mycoplasma* PJI was small, it still indicated that mNGS is a potential tool for the diagnosis of *Mycoplasma* PJI.

Although this method provided the reference for physicians, we should be cautious to interpret the results, as *Mycoplasma* required targeted antibiotic therapy that might cause adverse events. Therefore, further validation of the mNGS results through appropriate procedures was necessary. Currently, the effective isolation of pathogenic organisms remained a challenging problem for *Mycoplasma* PJI. Many papers had been reported various methods, including microbial culture, 16S PCR, mass spectrometer, etc. Xiang et al. (Xiang and Lu, 2019), Sneller et al (Sneller et al., 1986) and Muramatsu et al. (Muramatsu et al., 2022) reported a case of *Mycoplasma* PJI after joint arthroplasty, respectively. They all isolated *Mycoplasma* by routine microbial culture methods. The difference was that Xiang et al. (Wang et al., 2021) and Sneller et al. (Sneller et al., 1986) isolated *Mycoplasma* from blood agar, but Muramatsu et al. (Muramatsu et al., 2022), on the other hand, isolated pathogenic bacteria from the blood bottles. Notably, *Mycoplasma* can be easily missed because its small well-defined colonies on agar. In the present study, we used both blood agar and bottles for microbiological culture; however, pathogenic bacteria could not be isolated by these conventional microbiological culture methods. When the optimized culture method was applied, we successfully detected *Mycoplasma* in 4 cases. So, when mNGS is unavailable in some centers, the optimized culture methods could be taken into consideration. Rieber et al. (Rieber et al., 2019) reported a case of *Mycoplasma* PJI in a patient with normal immune function. This patient underwent total knee arthroplasty for osteoarthritis of the knee. Ten days before admission, urinary catheterization was performed due to prostatic hypertrophy and dysuria, which eventually led to disseminated *Mycoplasma* PJI. In this case, Rieber et al. (Rieber et al., 2019) successfully detected the pathogen by 16S PCR. 16S PCR is the earliest and most widely used in PJI pathogenic microorganism isolation, which could simply and quickly amplify the genes or fragments by specific or non-specific primers, and the amplified products could be sequenced, then the species of pathogenic bacteria could be obtained (Saglani et al., 2005; Fida et al., 2021). This was an effective tool for detecting *Mycoplasma* and widely used in recent years (Jensen et al., 2003; McAuliffe et al., 2005; Kim et al., 2022). However, this method could not effectively detect true pathogenic bacteria when there was mixed infection, and it could not distinguish contaminated bacteria from true pathogenic bacteria. In this study, we also used 16S PCR to detect *Mycoplasma*, but only successful in 5 patients. It was worth mentioning that *Mycoplasma* could be detected in case 2 (left knee), 3, 4, 6, 7 by 16s PCR, and failed in case 1 (mixed infection), case 2 (right knee, mixed infection) and case 5. This suggested the limitation of 16S PCR in mixed infection.

Based on previous reports and the results of this study, we proposed a standardized and optimized diagnostic procedure for *Mycoplasma* PJI, and all cases in this study were treated successfully after following this procedure. This procedure can also be used as a novel strategy for the diagnosis of PJI caused by other rare pathogenic bacteria.

This study had some limitations: 1) Although the largest number of *Mycoplasma* PJI cases were included in this study so far, the sample size was still far from adequate, so it was necessary to further expand the sample size by combining multiple centers to verify the true clinical value of this procedure; 2) Because *Mycoplasma* was difficult to culture and isolate, there might be other cases of *Mycoplasma* PJI that existed during the study, but were missed diagnosis due to the absence of mNGS test or negative mNGS results; 3) It was important to state that mNGS could only detected the nucleic acid of the microorganism, so it was impossible to determine whether the microorganism was alive or not. Therefore, it was necessary to consider and modify the antibiotic regimen according to the clinical reality.

## Conclusions

In conclusion, we reported the largest number of cases of *Mycoplasma* PJI and then summarize a mNGS based standardized and optimized procedure for the diagnosis of *Mycoplasma* PJI, which might also be offered as a new diagnostic strategy for rare pathogenic bacteria PJI.

## Data availability statement

The data presented in the study are deposited in the China National GeneBank Database (CNGBdb) repository, accession number CNP0001047; more details are available from the corresponding author on a reasonable request.

## Ethics statement

The studies involving human participants were reviewed and approved by The First Affiliated Hospital, Fujian Medical University. The patients/participants provided their written informed consent to participate in this study.

## Author contributions

WZ and XF designed the study; YQC, HD and YC performed the experiments and wrote the manuscript; CH, XC and CZ collected clinical samples and patient clinical information; ZH performed genotyping analysis; YH and WL analyzed data. All authors contributed to the article and approved the submitted version.
